# Effect on Nail Structure and Transungual Permeability of the Ethanol and Poloxamer Ratio from Cyclodextrin-Soluble Polypseudorotaxanes Based Nail Lacquer

**DOI:** 10.3390/pharmaceutics10030156

**Published:** 2018-09-11

**Authors:** Elena Cutrín-Gómez, Soledad Anguiano-Igea, M. Begoña Delgado-Charro, José Luis Gómez-Amoza, Francisco J. Otero-Espinar

**Affiliations:** 1 Department of Pharmacology, Pharmacy and Pharmaceutical Technology, University of Santiago de Compostela (USC), 15782 Santiago de Compostela, Spain; elenacutrin@hotmail.com (E.C.-G.); s.anguiano.igea@gmail.com (S.A.-I.); joseluis.gomez.amoza@usc.es (J.L.G.-A.); 2 Department of Pharmacy and Pharmacology, University of Bath, Bath BA2 7AY, UK; B.Delgado-Charro@bath.ac.uk

**Keywords:** transungual drug delivery, nail, medicated nail lacquers, onychomycosis, nail psoriasis ungueal, polypseudorotaxanes, methyl-β-cyclodextrin, poloxamers, ciclopirox olamine, clobetasol propionate

## Abstract

Aqueous-based nail lacquers have shown potential in promoting the diffusion of drugs into the nail. In our laboratory, we have recently developed a transungual delivery system based on an aqueous dispersion of cyclodextrin-poloxamer soluble polypseudorotaxanes, supramolecular host−guest assemblies that improves the drug permeation into the nail. However, the high-water content and the rheological and adhesive properties of this lacquer negatively affect properties that play a fundamental role in the patients’ acceptance such as stickiness, nail film formation or drying rate, properties. In this work, we have optimized the composition of these lacquers to improve these properties whilst maintaining good drug permeation profiles. Incorporating ethanol into the vehicle and reducing the proportion of Poloxamer 407 (PL), provided a good strategy. The use of hydro-ethanolic mixtures (>50% ethanol) and the reduction of the poloxamer concentration significantly improved the lacquer drying speed by reducing the stickiness and promoting film formation on the nail surface. Additionally, in a surprising way, the use of hydro-ethanolic vehicles further enhanced the permeation of ciclopirox olamine and clobetasol propionate, used for the treatment of onychomycosis and nail psoriasis respectively, into the nail and hooves.

## 1. Introduction

Research into new formulations to topically treat the ungual areas has grown in importance because nail diseases are becoming more frequent. Available treatments for these pathologies include oral antifungals, which are considered efficacious but have adverse effects and may cause drug interactions [1]. The few available topical therapies have low efficacy due to the relative low permeability of the nail [2,3,4,5].

The permeability of the nail plate depends on several factors, particularly nail hydration and swelling [6,7,8]. This was confirmed by Walters et al. [9] who discovered that a hydrated nail is more permeable to water than to alcohol and deduced that the nail plate behaves like a hydrogel. The same work demonstrated that diluted alcohols possessed a much higher nail permeability coefficient than some other alcohols, further confirming the role of water in facilitating ungual permeation. Later work demonstrated that ambient humidity increased the permeation of ketoconazole [10] as well as that of terbinafine [11]. Previously, we have investigated the effect of hydration on the microstructure of nails and bovine hooves, an animal model frequently used in in vitro ungual permeation studies [12]. We observed an increase in the porosity of the nails after treatment with water which led to greater in vitro permeation of triamcinolone. Therefore, nail hydration can be considered a simple tool with which to improve the penetration of drugs through the nails [13].

Most commercially available lacquers contain polymers dispersed or dissolved in organic solvents so as to increase the solubility of the drugs, and which lead to formation of water-impermeable films on the surface of the nails [14,15,16]. Examples of these formulations are Penlac^®^ that contains 8% ciclopirox dissolved in a mixture of ethyl acetate and isopropyl alcohol [17,18]; or Loceryl^®^ or Curanail^®^ that contain 5% amorolfine hydrochloride in a glycerol triacetate, butyl acetate, and ethanol mixed solution [19]. The use of these organic-based lacquers presents significant disadvantages such as irritation, poor drug delivery from the polymeric films and occlusive effects in fungal infections [14,20,21]. In addition, it has been suggested that alcohols and esters used as lacquer vehicles can increase the resistivity of the ungual barrier since they reduce the nail hydration and swelling [2,22,23,24]. Quintanar-Guerrero [25] demonstrated that ethanol, a known skin permeation enhancer, did not enhance the passage of antifungals into the nails. Further, more recent studies by Smith et al. [2] showed that ethanol, propylene glycol and polyethylene glycol 400 reduced the penetration and diffusion of ^3^H–water, ^14^C–urea, and ^14^C–tetraethylammonium ions into and across the nail and that the magnitude of the reduction observed was related to the organic solvent concentration.

To avoid the limitations of organic solvent-based lacquers, we have developed an aqueous formulation [26]. In a distinctive approach, this new water-based lacquer incorporated cyclodextrins, instead of organic solvents, to improve the poor water-solubility of the drugs [27,28]. Further, we demonstrated that soluble derivatives of β-cyclodextrins can act as nail penetration enhancers for antifungal and corticoids drugs [29]. Additionally, this lacquer incorporates Poloxamer 407 which forms soluble polypseudorotaxans with the β-cyclodextrins derivatives and the chemical enhancer N-acetylcysteine. These formulations provided good diffusion and penetration profiles for cyclopirox olamine and clobetasol propionate through combination of the hydrating and swelling effects due to water, the promoting effect of the cyclodextrins and acetylcysteine, and the increased solubility of the drug in the applied formulation. However, other aspects such as the stickiness, poor cosmetic appeal, and the drying speed of the applied formulation, required improvement to provide greater convenience of use and increased patients’ adherence. Formation of a film on the nail surface with adequate adhesiveness and drying properties can be promoted by inclusion of mixtures of volatile solvents like ethanol or ethyl acetate and water in the vehicle. These organic solvents are rapidly evaporated upon application promoting formation of a smooth, hydrated and unsticking layer. Nevertheless, as described above, their presence in the lacquer can lead to decreased performance of the formulation and, therefore, a balance must be found for a suitable composition that provides efficient drug delivery without compromising the convenience of use and cosmetic appeal of the formulation. This task is not simple given that the role played by the cyclodextrins, poloxamer 407 and *N*-acetylcysteine in ensuring drug solubility and efficient permeation could be modified by the addition of ethanol to the formulation.

In this context, the objective of this study was to evaluate the influence of formulation changes, in particular the inclusion of ethanol and the proportion of poloxamer, in the cosmetic appeal, convenience of use, and performance as ungual drug delivery systems of cyclodextrin based soluble polypseudorotaxans nail lacquers containing antifungal and antipsoriatic drugs.

## 2. Materials and Methods

### 2.1. Materials

Bovine hooves were obtained from Compostelana de carnes S.L, a local slaughterhouse nearby Santiago de Compostela, Spain. The hooves were cleaned with water and soaked for 24 h to facilitate their cutting in small slices (Ufesa Professional Slicer FS50, 0.3–0.7 mm thick). The slices were dried at room temperature for 48 h before being used in the experiments.

Finger and toe nail clipping samples were donated by healthy volunteers from both sexes, aged between 25 and 65 years old after signing informed consent and the nail donation protocol was approved by the Ethic Committee of Galicia (Project identification code: 2018/099. Date of approval 22 February 2018). The nails clips were cleaned with water, and dried at room temperature for 48 h. Finally, samples were stored in a glass container at room temperature until use. The samples used for Mercury intrusion porosimetry studies measured 1–3 mm and the nails clips used for permeation studies had a length greater than 8 mm.

Components of the lacquer included Poloxamer 407 (PL, Sigma-Aldrich-Merck, Darmstadt Germany), methyl-β-cyclodextrin (degree of molar substitution 0.57 and molecular weight 1191 Da, Crysmeb, Roquette Laisa, Valencia, España), acetylcysteine (Acorfarma, Madrid, Spain), ethanol (Merck Millipore, Darmstadt, Germany), ciclopirox olamine (Fagron Iberica, Tarrasa, Barcelona, Spain), clobetasol-17-propionate (Acorfarma, Madrid, Spain) and purified water (Elix^®^, Merck-Millipore, Darmstadt, Germany).

Phosphate-buffered saline (PBS) was prepared from sodium dihydrogen phosphate dodecahydrate, potassium dihydrogen phosphate and sodium chloride according to the 8th edition of the European Pharmacopoeia (analytical grade from Merck, Darmstadt, Germany). Sodium azide (Panreac Quimica SA, Barcelona, Spain), 30 mg/L, was added to PBS to prevent microbial growth. Ciclopirox olamine and clobetasol propionate were extracted from the nails after penetration studies using methanol from Prolabo (Spain) 

OnyTec^®^ nail lacquer (Reig Jofré S.A., Barcelona, Spain) containing 80 mg/g of ciclopirox was used as a reference product in the permeation assays.

Uñabase^®^ nail Lacquer (Fagron Iberica, Barcelona, Spain) was used as vehicle to prepare the reference lacquer of Clobetasol propionate.

### 2.2. Mercury Intrusion Porosimetry (MIP)

These experiments aimed to determine whether the presence of ethanol alters the microstructure of the nail plate. Healthy nail samples were immersed in either an aqueous solution or a 1:1 hydro-alcoholic solution for 24 h. Next, they were removed from the solution and the water was eliminated. For this, the samples were frozen in liquid nitrogen as to maintain their structure and immediately freeze-dried (Telstar LyoQuest Plus lyophiliser, Telstar, Terrassa, Spain). After freeze-drying, approximately 0.6 g of grouped nails (depending on size, the number of nail clippings combined for one experiment could range between 50–60), were placed in a 3 mLpenetrometer and analysed in a pressure interval of 0.004 to 172.4 MPa, using a Micromeritics Autopore IV porosimeter (Norcross, GA, USA). The pore size distribution structure and the water permeability were modelled using the PoreXpert 1.3 software (Environmental and Fluid Modelling Group, University of Plymouth, Plymouth, UK). PoreXpert is a specialized software that uses mercury porosimetry data to generate 3D void structures of porous material with similar percolation properties [12,29].

### 2.3. RAMAN Spectroscopy

Samples of healthy nails of approximately 1–2 mm were submerged for 24 h in an aqueous solution or in a 1:1 hydroalcoholic solution and freeze-dried. Then, Raman spectra of the samples were obtained in triplicate using a random scanning of the top and bottom surfaces of the nail samples with a Raman FT-Bruker Raman Scope spectrometer (Bruker Biosciences Espanola S.A, Madrid, Spain).

### 2.4. Preparation of the Lacquers

The starting composition of the lacquers was based on our prior work [26]. In the current work, the proportion of Poloxamer 407 and the composition of the vehicle were modified by partial substitution of water by ethanol (Table 1).

Briefly, the preparation involved the following steps: 10% of MBCD was dissolved in water and water–ethanol mixtures (1:1; 1:2; 1:3 and 1:4), under constant stirring and at a temperature of about 4 °C to facilitate dispersion of the poloxamer 407 which was incorporated in different concentrations once the cyclodextrin was dissolved. 10% *N*-acetylcysteine was added keeping the same temperature and constant stirring conditions. Finally, either ciclopirox olamine or clobetasol propionate were added in sufficient amount to ensure saturation of the vehicle. Next, the suspension was continuously stirred at room temperature for 24 h and filtered through 0.45 μm nylon membrane filters (Merck Millipore, Darmstadt, Germany) to remove the excess undissolved drug. The concentration of ciclopirox olamine was determined by ultraviolet spectroscopy (Hewlett-Packard 8452A, Hewlett-Packard Española, S.A., Madrid) and that of clobetasol propionate, by liquid chromatography using UPLC QSM Waters Acquity chromatography equipment connected to a Tandem Xevo TQD mass detector (Waters^®^, Saint-Quentin, France).

Ciclopirox olamine was loaded in lacquers at saturation and solubility of the different composition are discusses in result and discussion section. In the case of the 5% PL water:ethanol 1:1 lacquers the ciclopirox olamine and Clobetasol propionate loaded was 21.3 ± 2.08 mg/mL and 1.6 ± 0.23 mg/mL respectively.

The reference lacquer containing 8% of Clobetasol propionate was prepared by addition of 0.8 g of drug in 10 mL of the Uñabase^®^ under continues agitation, until complete dissolution.

### 2.5. Cosmetic Appeal

To evaluate the cosmetic appeal, the drying time of the lacquer and the stickiness were evaluated. To do this, 500 μL of the lacquer were poured onto a black background plate. The time required for the applied film to be completely dried, that is, until it was no longer sticky was recorded.

### 2.6. Release Studies of Ciclopirox Olamine from Nail Lacquers

Drug release experiments were carried out using vertical Franz cells, with an effective diffusion surface of 0.79 cm^2^. In the donor compartment, 2000 μL of the formulation was deposited and, in the acceptor compartment, a phosphate-buffered saline solution of pH 7.4 (Pharm. Eur) was incorporated and maintained at 37 °C under continuous stirring. The two compartments were separated by a MWCO > 12,000–14,000 Da dialysis membrane (Visking dialysis membrane, Medicell Membranes Ltd., London, UK). Samples were collected from the acceptor at stipulated time intervals, and the same volume replaced with fresh PBS. The ciclopirox olamine concentration was determined spectrophotometrically at 308 nm (Hewlett Packard 8452 Diode-array spectrophotometer, Hewlett-Packard Española, S.A., Madrid).

The amount of ciclopirox released vs. time (*t*) profiles were fitted to a zero order and a diffusion kinetics according to the following equations:

Zero Order: Released amount = k*t*

Diffusion: Released amount = k√*t*

The analysis was done using the non-linear regression module of the software GraphPad Prism 6.01 (GraphPad Software, La Jolla, CA, USA).

### 2.7. Drug Permeability and Penetration Studies on Nails and Hooves

Due to the difficulty in sourcing samples of human nails of adequate size to carry out diffusion studies, bovine hooves were used as a model in the first stage of the study. Hooves have been used by different authors as a substitute of the nail [30,31] and allowed a first screening of the formulations. Subsequently, the performance of those lacquers providing the highest drug permeation across hooves was verified in permeation tests using human nail samples.

Permeation experiments used the same methodology described above for the release test experiments but used either hoof slices or nails clips placed between two cylindrical Teflon^®^ adapters (Mecanizados del Noroeste, Santiago de Compostela, Spain) instead of the dialysis membrane.

Hoof slices and nails were hydrated in water for two hours before the experiment. Once hydrated, the thickness of the slices was measured with a micrometer (Mitutuyo, Kawasaki, Japan) and samples were selected among those having similar thickness (0.4–0.7 mm) and not presenting defects or cracks. The Teflon adapters provided an effective diffusion area of 0.196 cm^2^ and the ensemble (nail/hoof slice—adaptor) was clamped between the compartments of the Franz vertical diffusion cells.

A volume of 2 mL of the lacquers was added to the donor compartment. 30 mg/L of sodium azide in PBS (to prevent microbiological and algae growth) or a dissolution of a 5% HPB with sodium azide (30 mg/L) was used as receptor media to assure sink conditions, for ciclopirox olamine and the clobetasol propionate respectively.

The permeation test took over a period of 11 days. 1 mL samples were taken from the receptor every 24 h and replaced with fresh PBS.

At the end of the diffusion study, the amount of drugs inside the hoof and nails was determined. Nails and hooves were removed from the adapter and cleaned with distilled water. The area exposed to the lacquer was cut, divided into small fragments, weighed, and incubated in 5 mL of phosphate buffer containing 5% of methanol for 6 days at 25 °C, under moderate stirring.

The drug concentration in the receptor was quantified after filtration of the samples through nylon filters (0.22 µm, MERCK MILLIPORE) and dilution 1:1 with NaOH 1 M. Ciclopirox was quantified by at 308 nm UV spectroscopy (Hewlett Packard 8452A) [12,31,32]. Clobetasol propionate quantification was performed on a MS/MS tandem Waters Xevo^®^ TQD detector linked to an Acquity UPLC^®^ H-Class system (Waters^®^, Saint-Quentin, France) using TargetLynxTM Application Manager. For quantification A BEH C18 Acquity column of 2.1 × 50 mm and particle size of 1.7 µm was used (Waters^®^, Czech Republic). The column was maintained at 40 °C and isocratic conditions (water:methanol 20:80) and a flux of 0.5 /mL min were used. The volume of injection was 5 µL and the mass spectrometric data acquisition was performed by positive electrospray ionization, in multiple reaction monitoring (MRM) mode. Regarding quantification, the detection conditions were: ion transitions of *m*/*z* 467.1 > 355.1 (Cone voltage 100 V, collision energy 10 V), cone gas 80 L/h, desolvation gas flow 1100 l/h, capillary voltage of 0.55 kV, desolvation temperature 450 °C and source temperature 146 °C.

## 3. Results and Discussion

To study nail microstructural changes caused by the different investigated vehicles, we have used mercury intrusion porosimetry (MIP) followed by data analysis and modelling of the microporous structure using the PoreXpert^®^ software.

**Figure 1:**
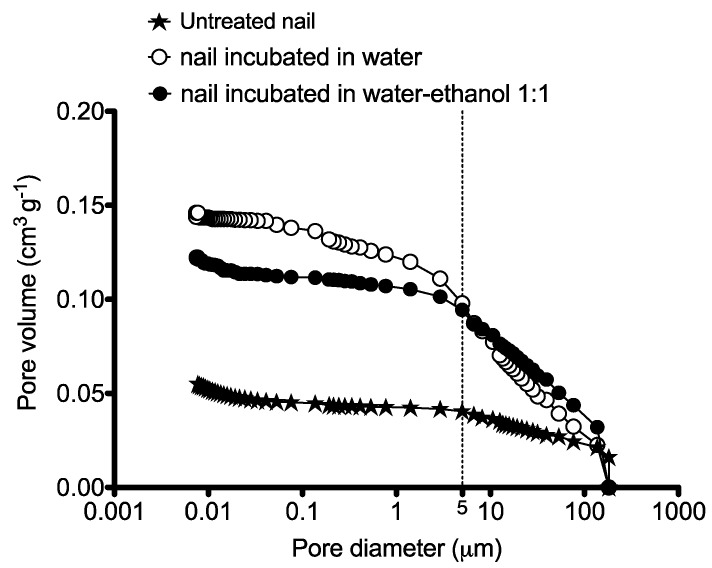
Cumulative curves of porous volume obtained by MIP for non-incubated nails and for nails incubated in water or in a 1:1 hydroalcoholic mixture.

The MIP size distribution of the internal pores corresponding to the untreated nails, nails incubated in water and in a 1:1 hydroalcoholic solution are shown in Figure 1. The total porosity of the nails (Table 2) increased 2.2 and 1.8-fold following 24 h incubation in water and hydro-alcoholic solution, respectively. The specimens treated with the hydroalcoholic mixture showed a total porosity of 12.8% that was slightly lower than that of the nails incubated with water (15.24%).

In addition to total porosity, it is also important to consider pore size distribution. Figure 1 shows that nails incubated in the hydroalcoholic mixture had a higher proportion of pores with a >5 μm diameter than those incubated in water. This situation is reversed when pores with diameters smaller than 5 μm are taken into consideration. The results suggest that different solvent mixtures will affect the overall porosity as well as the pore size distribution, with potential consequences in solvent penetration patterns and hence in drug diffusion processes.

Based on the pore size distributions obtained by PIM, the nail structures were analyzed and modelled using the PoreXpert^®^ software (V1.3, Environmental and Fluid Modelling Group, University of Plymouth, Plymouth, UK). This software generated a three-dimensional void structure that had the same percolation characteristics as the material experimentally characterized by PIM. A full description of this approach can be found in [12].

**Figure 2:**
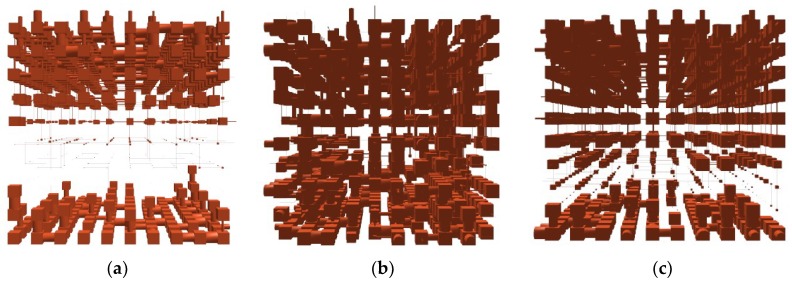
Models of the structures made with PoreXpert^®^ software for the untreated nail (**a**) and the nails incubated in water (**b**) or in 1:1 hydro–alcoholic mixture (**c**).

Figure 2 shows the structures of the unit cell resulting from the modelling of untreated nails and of those incubated in the 1:1 hydroalcoholic solution and in water. In these structures, the pores of the nails are represented by cubic spaces interconnected by cylindrical throats, the same geometry assumed by PIM for calculating the distribution of pore size.

The model of untreated nails showed numerous pores inter-connected at the superficial level of the structure. However, as we move inward (from the fifth layer of the model) a very compact and non-porous area is found, which represents an important barrier to the diffusion of solvents and larger molecules, such as drugs. Another information resulting from the analysis is the correlation level values shown in Table 2. The correlation level ranges from 0, when the structure is randomized, to 1 when the unit cell is a perfectly organized structure [12,29]. Thus, the 0.692 correlation level assigned to the model structure corresponding to the healthy nail (Table 2) indicates that this is an ordered structure and further, the value is consistent with the presence of well-defined areas (i.e., internal and superficial) with significantly different porosity.

Hydration modified the pore distribution in the nail plate by hydrating and swelling the keratin structure [12,19]. The model structure for the nails incubated in water exhibits a more porous inner area with a random pore size distribution, and this is reflected in a lower correlation level value (0.159, Table 2).

The presence of ethanol in the 1:1 hydroalcoholic mixture lessens the effect just described. Compared to pure hydration, this treatment resulted in formation of a slightly less porous structure, especially in the innermost and most ordered part of the nail. Figure 2c shows a progressive reduction of the pore size towards the inner part of the structure, and for this reason, the model correlation level value (0.709, Table 2) is higher and closer to that obtained with healthy, untreated nails.

**Figure 3:**
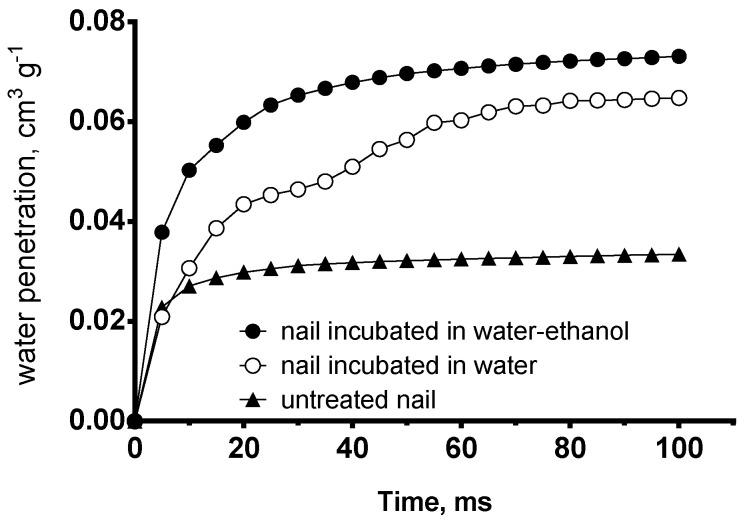
Rate of water penetration into the structural models corresponding to untreated nails and nails incubated in water and in water-ethanol 50% predicted by PoreXpert^®^ models. Water penetration is expressed as cm^3^ of water penetrated into one gram of nail.

Finally, water nail permeability expressed as the volume of water penetrated in one gram of the nail vs. time (Figure 3) was estimated based on the structural models obtained using PoreXpert^®^.

The prediction suggests that the fastest water uptake occurs through the structure corresponding to nails incubated in 1:1 water:ethanol. In this case, the penetration rate quickly reaches a plateau as it is the case for untreated nails. However, for the latter, water uptake is much lower as expected from a less porous system. Finally, in the case of nails incubated with water, the rate of uptake increases more gradually, taking longer to reach a plateau.

This prediction reveals that solvent uptake into a porous structure is a complex phenomenon; the rate with which a solvent gets incorporated into the structure depends on the size distribution of the pores and to the connectivity existing between pores. Nails incubated with the hydroalcoholic mixture have a higher pore volume and connectivity in the upper layers of the model but the pore size decreases progressively towards the inside of the nail. Such a structure could explain the rapid filling of water into the upper area, and a quick diffusion inside the nail promoted by the capillary forces. On the contrary, nails hydrated with water have a more random distribution of pores throughout the whole structure, which provides a more gradual solvent capture because some of the internal pores with largest sizes can act as reservoirs.

**Figure 4:**
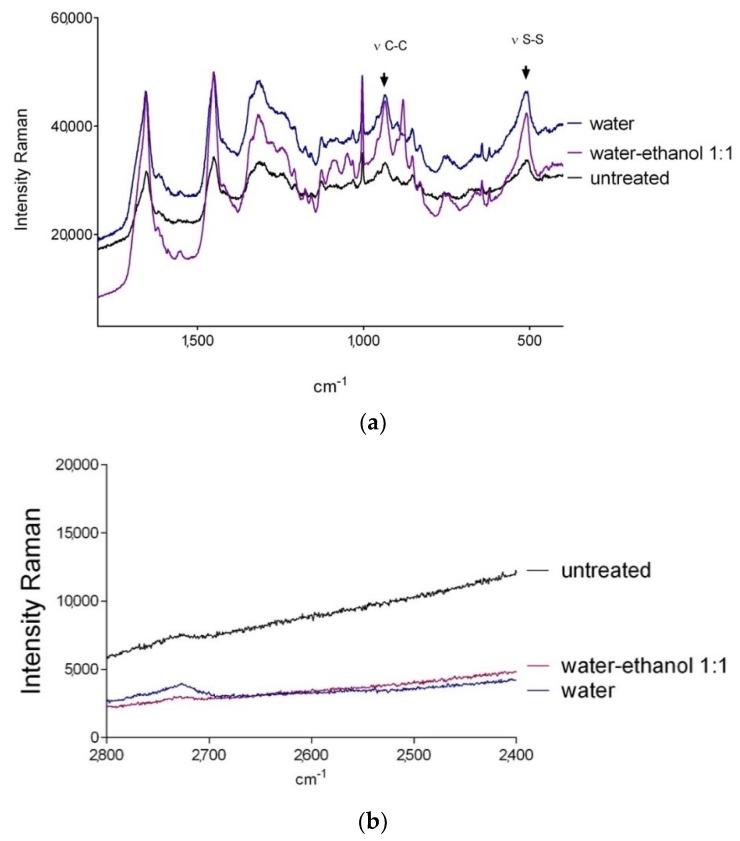
RAMAN spectra of the external surface of the three nail samples studied in an interval of 400–1800 cm^−1^ (**a**) and 2400–2800 cm^−1^ (**b**).

The swelling of protein materials depends largely on the alterations caused by the uptake of the solvents. The mechanical properties of the nail plate are primarily determined by a high content of structural proteins such as keratins the integrity of which is very much determined by the presence of disulphide bonds. Therefore, to investigate alterations in the disulphide bonds of the keratin, we have used RAMAN spectroscopy. Figure 4 shows the Raman spectra obtained after randomly scanning the surface of the nails on its external side in an interval between 400–1800 cm^−1^ (Figure 4a) and between 2400–2800 cm^−1^ (Figure 4b). All the samples presented a spectrum characteristic of protein structures [33]. In Figure 4a the assignment of the characteristic signals of the chemical bonds in keratins has been included. As discussed above, the disulphide bridges are especially relevant so the ratio of its area with respect to that of the C–C bonds was determined. These normalized ratios facilitate sample comparison; and their values 1.1 ± 0.15; 1.0 ± 0.12 and 0.94 ± 0.13 for the untreated nail, the hydrated nail and the nail incubated in a 1:1 hydro alcoholic mixture respectively, were not significantly different.

In addition, the lack of signal corresponding to the –SH groups at 2550–2600 cm^−1^, which originate from the rupture of the disulphide bonds, is particularly informative. The absence of this band in all cases (Figure 4b) further supports that neither water nor the 1:1 water:ethanol mixture had a significant effect on the keratin disulphide bridges and that the increase in nail porosity observed (Figure 1) was not due to the rupture of these bonds.

As mentioned above, one of the objectives of this study was to improve the poor cosmetic properties and convenience of use of the original formulation, especially with regard to the drying speed and the stickiness of the film formed by the lacquer on the nail upon application, as this would facilitate patient adherence. For this purpose, we have studied how the drying time was influenced by the incorporation ethanol and Poloxamer 407 in different proportions with the results summarized in Table 3.

**Figure 5:**
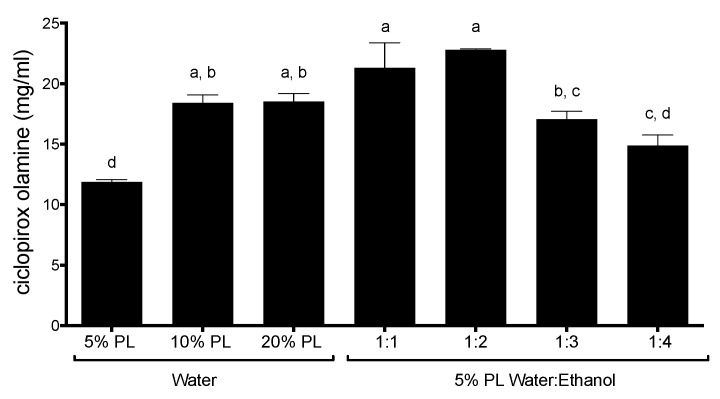
Solubility (mean ± SD, *n* = 6) of ciclopirox olamine in different aqueous lacquer bases made with different proportions of Poloxamer 407 and in hydroalcoholic lacquers made with different proportions of ethanol and with 5% Pluronic^®^ F127. Same letter indicates homogeneous groups (one-way ANOVA, Tukey post-hoc test, α < 0.05).

The reduction of Poloxamer 407 concentration at 5% shortened the drying time to less than 10 min, as well as reducing stickiness. Thus, this content of Pluronic was retained to investigate the effect of the incorporation of ethanol which, as expected, reduced the drying time to 5 min for the water:ethanol ratios of 1:1 to 1:3 or, even to just 3 min with highest ratio (1:4) of ethanol tested. Consequently, by reducing Poloxamer 407 concentration and increasing the ethanol ratio in the vehicle better drying properties were obtained. On the other hand, changes in the solvent composition can impact the drug loading capacity and release properties of the lacquers. In these relatively complex vehicles the Poloxamer 407 interacts with the methylated cyclodextrins by forming soluble polypseudorotaxanes [12], resulting in a complex system in which the micelles of the polymeric surfactant, the cyclodextrin-drug inclusion complex, and the polypseudorotaxanes coexist. Thus, a series of interactions and competitions are established between the different components that make it difficult to predict the impact of compositional changes on the drug solubility. Thus, an experimental approach was taken, and Figure 5 shows the maximum amount of ciclopirox olamine solubilized by the lacquers. Larger Poloxamer 407 concentrations facilitated the solubilization of the drug, with an increased Pluronic concentrations of 10–20% compared to the one prepared with 5% (one-way ANOVA, Tukey’s multiple comparison test, α < 0.05). However, no significant differences were found between the 10% and 20% Poloxamer 407 lacquers.

The incorporation of alcohol in the lacquers allows the maximum amount of ciclopirox olamine that can be dissolved in the lacquers to increase moderately, obtaining the best results when using ratios of 1:2 water–ethanol with which solubilities of ciclopirox higher than 20 mg/mL (α < 0.05) are obtained.

**Figure 6:**
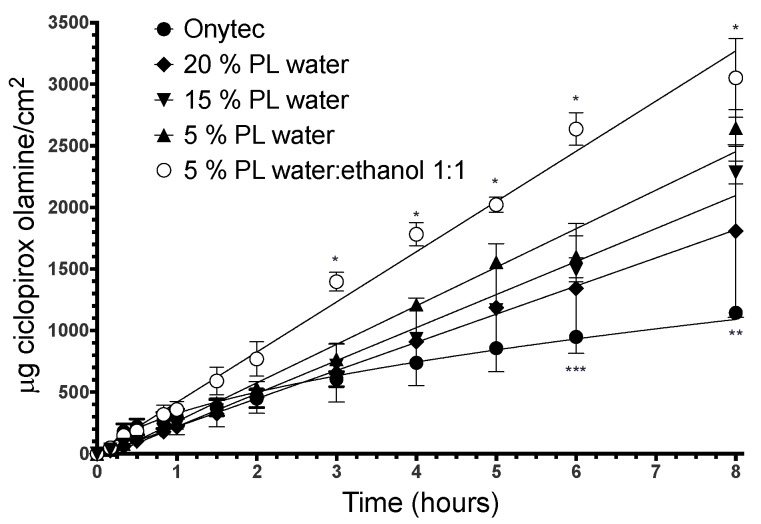
Release profiles (mean ± SD, *n* = 6) of aqueous lacquers made with different proportions of Poloxamer 407 and of the lacquer made with a 1:1 water–alcohol mixture and 5% poloxamer 407. * indicates significant differences between 5% PL water:ethanol 1:1 and the rest of lacquers (α < 0.05); ** indicates significant differences between OnyTec^®^. and the rest of lacquers (α < 0.05); *** indicates significant differences between OnyTec^®^ and 5% PL water.

Figure 6 presents the release profiles of ciclopirox olamine from the different lacquers. The results show that there are hardly any differences between aqueous lacquers made with different proportions of Poloxamer 407, despite differences in the drug load. Nevertheless, significant differences (two-way ANOVA, factors: Time and formulation, Tukey post-test, α < 0.01) were observed in the CPO released between the 1:1 water–ethanol lacquer and the aqueous and reference Onytec^®^ lacquer after 3 h and between aqueous lacquer and Onytec^®^ after 6 h.

This behavior indicates that aqueous lacquers allow for a good control of the release of ciclopirox, a process that conforms to zero order kinetics (Table 4). This mechanism of release may be related to the delivery and transfer of the drug from the complex gel formed by the polymeric micelles, the inclusion complex and the polypseudorotaxanes.

Initially, aqueous lacquers release the drug in a similar manner to the commercial formulation Onytec^®^. On the contrary, at later times, aqueous lacquers release more ciclopirox (Table 4).

The formulation with the highest release rate is the one containing 5% Poloxamer 407 and incorporating a water:ethanol mixture at a 1:1 ratio. This higher release rate is due to the higher ciclopirox load in the formulation and to the decrease in the polarity of the mixture, which facilitates delivery to the release medium by reducing the affinity of the drug for the different components of the vehicle.

The next stage of this work investigated the influence that changes in lacquer composition had on drug penetration and diffusion through the nail. A first screening of the formulations performance used a bovine hoof model and, in a second step, the most promising lacquers were tested with human nail clippings. Given the limitations in sourcing human diseased nails, bovine hooves have been used by different researchers to model the nail in in vitro permeation experiments [12,24,29,30,31,34,35,36,37]. Whilst it is known that bovine hoof is more porous and permeable than healthy human nails, correlation between the permeation measured across the two membranes has been reported [12,24,31] thus suggesting that trends observed with hooves will be predictive of those to be found with nails. On the other hand, healthy human nails have a lower porosity than diseased nails [32]. It is expected that delivery performance through diseased nails will be somewhere between the two models and that will be comparable to that observed in human nails as the disease recedes. Thus, it seems reasonable to use bovine hooves, easily sourced, for in vitro permeation studies aiming to screening the most promising formulations [34] and then verify the potential of the best performing vehicles with healthy human nails.

**Figure 7:**
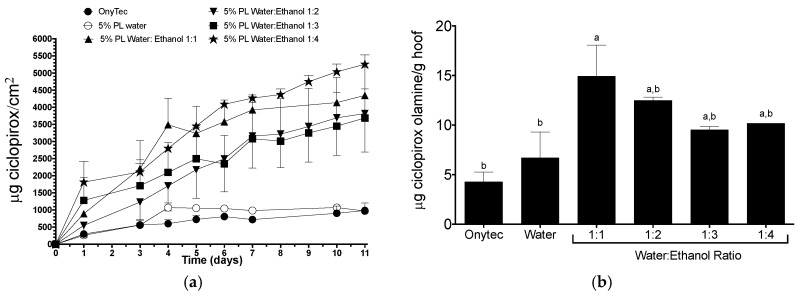
(**a**) Cumulative permeation (mean ± SD, *n* = 3) of ciclopirox olamine across bovine hoof. (**b**) amount of drug recovered from the hoof after eleven days. All the of the lacquers were made with 5% Poloxamer 407 and water:ethanol mixtures in the ratios indicate, Onytec^®^ (containing 8% ciclopirox) was used a reference. Same letter indicates homogeneous groups (one-way ANOVA, Tukey post-hoc test, α < 0.05).

The permeation profiles of ciclopirox olamine across hoof and the amount of drug recovered from the hoof slices after an eleven-day test are shown in Figure 7. It can be observed that the lacquer made with water and the reference (Onytec^®^) product provided similar diffusion profiles. In contrast, ciclopirox olamine diffusion was much larger when delivered from lacquers were based on hydroalcoholic solutions; there was no clear effect of the specific level of alcohol in the mixture. A two-way ANOVA and Tukey post-test showed that the from the third day of the study, the amount of drug permeated across the hoof when delivered from hydroalcoholic-based lacquers was significantly different than when delivered from the reference product and the aqueous lacquer (α < 0.01). No significant differences were observed between the solutions prepared with different proportions of ethanol.

The amount of ciclopirox olamine recovered from the hoof after the eleven days permeation test was also greater for the hydroalcoholic based lacquers than for the reference product and the aqueous based lacquer (α < 0.05).

**Figure 8:**
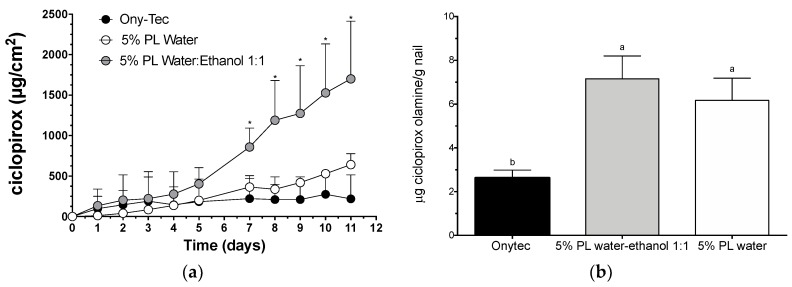
(**a**) Cumulative permeation of ciclopirox olamine across human nail (mean ± SD, *n* = 3). (**b**) amount of drug recovered from human nail clippings after an eleven days permeation study. The lacquers tested contained 5% Poloxamer 407 and either water or a 1:1 water:ethanol mixture. Onytec^®^ was used a reference. Stars indicates significant differences with the other lacquers (two-way ANOVA, Tukey post-hoc test, α < 0.05) and same letter indicates homogeneous groups (one-way ANOVA, Tukey post-hoc test, α < 0.05).

Given the improvement in the drying time and in the diffusion and penetration of CPO through the hoof provided by the lacquers prepared with 5% Poloxamer 407 in hydroalcoholic vehicles, the lacquer made with 1:1 water:ethanol vehicle was selected to be tested on human nails. This lacquer was compared to the marketed product Onytec^®^ and the lacquer containing water as vehicle and the results are shown in Figure 8. The ungual fluxes of CPO calculated for OnyTec^®^ and for the 5% PL water lacquer were 57.35 ± 3.67 µg cm^−2^ day^−1^ (0–11 days, *R*^2^ = 0.9644) and 16.72 ± 3.44 (0–11 days, *R*^2^ = 0.7241) µg cm^−2^ day^−1^, respectively. The results were superior for the 5% PL 1:1 water:ethanol lacquer: 70.48 ± 8.49 (0–5 days, *R*^2^ = 0.9451) or 216.3 ± 12.19 µg cm^−2^ day^−1^ (5–11 days, *R*^2^ = 0.9874). A two-way ANOVA found that both the formulation used and the test time had a significant effect on the drug permeation; the multiple comparison Tukey test showed that the amount of ciclopirox delivered from the 1:1 water–ethanol based lacquer was greater than that delivered from the OnyTec^®^ and 5% PL water lacquer from the fifth day of the test onwards. Briefly, also in the case of human nails, the lacquer with the 1:1 water-ethanol vehicle provided much more delivery of CPO (diffusion across the plate and penetration into the plate) than the aqueous-based lacquer and by the reference lacquer Onytec^®^ (α < 0.05).

Finally, we investigated whether these results could be extrapolated to other drugs of interest in ungual delivery. For this, the same lacquer (5% PL 1:1 water:ethanol) was used as a potential topical formulation for clobetasol propionate, a drug used to treat nail psoriasis. No commercial formulations of this drug are available so a compounding formulation dispensed by dermatologists for this indication was used as a control. This formula is prepared by dispersing the drug at an 8% *w*/*w* concentration in an organic nail lacquer base; in this work the lacquer base Uñabase commercialized by FAGRON Ibérica was used. Differently to this compounding formulation, the clobetasol loading in the 5% PL 1:1 water:ethanol lacquer was much lower, 1.6 ± 0.23 mg/mL.

**Figure 9:**
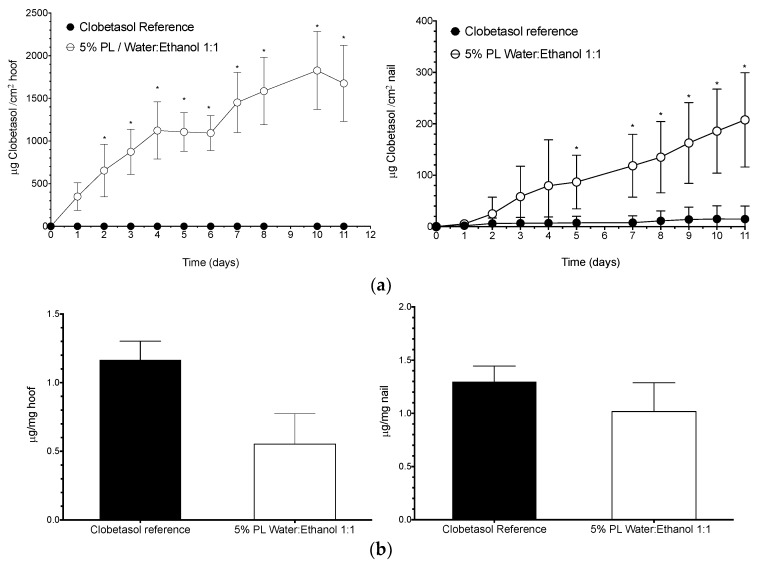
(mean ± SD, *n* = 3) (**a**) Top panels: Permeation profiles of clobetasol propionate and (**b**) Bottom panels: Amount of drug recovered from nail or bovine hoof after the eleven days of permeation study. In both cases, the left panels show the results for bovine hoof and the right panels the results obtained with human nail clippings. In both models, the lacquer made with 5% Poloxamer 407 and a 1:1 water:ethanol mixture and the reference lacquer containing 8% of clobetasol propionate in Uñabase (see text and Material and Methods) were compared. Stars indicates significant differences with the other lacquers (two-way ANOVA, Tukey post-hoc test, α < 0.05).

Delivery (diffusion and accumulation) of clobetasol propionate into bovine hoof and human nail (Figure 9) during the eleven test-days from the reference lacquer was practically negligible. However, the hydroalcoholic based lacquer provided significant drug delivery into and across the hoof, with fluxes quite similar to that obtained for triamcinolone acetonide in previous studies with aqueous based lacquers [12]. A similar trend was observed for the human nail, although the permeation fluxes observed were lower than measured with the animal model. These differences between nail and hoof are as expected since the hoof structure is more permeable to drugs than the nail [31]. The lacquer elaborated with 5% Poloxamer 407 and the 1:1 water:ethanol mixture provided a continuous flux of clobetasol of 20 μg/cm^2^ into the nail during the eleven days of testing.

In terms of penetration values, values of 0.55 μg/mg for hoof and 1.02 μg/mg for nail were obtained with the 1:1 water-ethanol lacquer, a lower recovery than that determined for ciclopirox olamine but clearly higher than the 0.27 μg/mg obtained for triamcinolone acetonide in the nail [31]. Briefly, whilst the delivery of the corticosteroid was lower than for the antifungal, it was much optimized when compared to previous work that reported no triamcinolone acetonide through the nail [31]. Given the high potency of clobetasol, this delivery might be enough to be of therapeutic benefit; unfortunately, there is not information about the concentration in the nail required to obtain acceptable pharmacological response.

Finally, it should be noted that the results obtained with the reference lacquer must be taken with caution since the process of washing of nails and hooves prior to the extraction of the drug is performed with water, which effectively eliminates the hydroalcoholic lacquers. However, we were not able to eliminate the hydrophobic film from the surface of the hoof formed after the application of the organic lacquer. Consequently, the value determined in this case includes the sum of the penetrated drug and the remnant of the lacquer that remains on the surface.

## 4. Conclusions

The incorporation of ethanol and the reduction of the proportion of Poloxamer 407 in the aqueous lacquer of soluble polypseudorotaxans of poloxamer and soluble β-cyclodextrins derivatives provided unexpected results. The lacquer-drying speed was improved by increasing the ungual permeability of ciclopirox olamine and clobetasol propionate.

The data suggest that an incorporation of ethanol increased the dose of ciclopirox olamine incorporated in the lacquer without apparently affecting the hydration of nail and improving their spreading and humectation. Also, a ratio of 5% of Poloxamer 407 is adequate to obtain a quick drying time and a good drug nail penetration.

Based on the results, the lacquer prepared with 10% methyl-β-cyclodextrin, 5% Poloxamer 407 and a 50% hydroalcoholic solution have high potential as a vehicle to prepare medicated nail lacquers.

## Figures and Tables

**Table 1:** Composition of the lacquers used in this work. Quantities are expressed in % *w*/*v* for 10 mL of lacquer.

Component	20% PL Water	10% PL Water	5% PL Water	5% PL Water:Ethanol 1:1	5% PL Water:Ethanol 1:2	5% PL Water:Ethanol 1:3	5% PL Water:Ethanol 1:4
Poloxamer 407	20%	10%	5%	5%	5%	5%	5%
MBCD	10%	10%	10%	10%	10%	10%	10%
N-acetylcysteine	10%	10%	10%	10%	10%	10%	10%
Water	10 mL	10 mL	10 mL	5 mL	3.3 mL	2.5 mL	2 mL
Ethanol	-	-	-	5 mL	6.7 mL	7.5 mL	8 mL

**Table 2:** Porosity of treated and control human nail clippings and correlation values obtained by PIM and ensuing data analysis and modelling with PoreXpert software.

Nail Treatment	Porosity (%)	Correlation
Untreated nail	6.98	0.692
Nail in water	15.24	0.159
Nail in water–ethanol	12.80	0.709

**Table 3:** Influence of the proportions of Poloxamer 407 and ethanol in the drying times of the developed lacquer bases. In all cases, the lacquers contained 10% MBCD and 10% NAC.

Poloxamer 407 (%)	Water:Ethanol	Drying Time (min)
5	1:0	<10
10	1:0	>10
20	1:0	>10
5	1:1	<5
5	1:2	<5
5	1:3	<5
5	1:4	<3

**Table 4:** Result of adjusting the results of the release of ciclopirox from lacquers to zero order and diffusion kinetics. (Average ± standard error, *n* = 3).

Parameter	5% PL Water	10%PL Water	20% PL Water	5% PL Water-Ethanol 1:1	Onytec
*k* (µg/cm^2^·h)	306.0 ± 49.1	278.9 ± 53.4	228.5 ± 7.6	424.7 ± 27.7	-
*R* ^2^	0.9772	0.9679	0.9990	0.9962	-
*k* (µg/cm^2^·h^0.5^)	-	-	-	-	417.6 ± 23.1
*R* ^2^	-	-	-	-	0.9866
